# Linear Mathematical Model for Seam Tracking with an Arc Sensor in P-GMAW Processes

**DOI:** 10.3390/s17030591

**Published:** 2017-03-14

**Authors:** Wenji Liu, Liangyu Li, Ying Hong, Jianfeng Yue

**Affiliations:** School of Mechanical Engineering, Tianjin Polytechnic University, No. 399 Bin Shui Xi Road, Xi Qing District, Tianjin 300387, China; liliangyu@tjpu.edu.cn (L.L.); hongying@tjpu.edu.cn (Y.H.); yuejianfeng@tjpu.edu.cn (J.Y.)

**Keywords:** arc sensing, P-GMAW, mathematical model

## Abstract

Arc sensors have been used in seam tracking and widely studied since the 80s and commercial arc sensing products for T and V shaped grooves have been developed. However, it is difficult to use these arc sensors in narrow gap welding because the arc stability and sensing accuracy are not satisfactory. Pulse gas melting arc welding (P-GMAW) has been successfully applied in narrow gap welding and all position welding processes, so it is worthwhile to research P-GMAW arc sensing technology. In this paper, we derived a linear mathematical P-GMAW model for arc sensing, and the assumptions for the model are verified through experiments and finite element methods. Finally, the linear characteristics of the mathematical model were investigated. In torch height changing experiments, uphill experiments, and groove angle changing experiments the P-GMAW arc signals all satisfied the linear rules. In addition, the faster the welding speed, the higher the arc signal sensitivities; the smaller the groove angle, the greater the arc sensitivities. The arc signal variation rate needs to be modified according to the welding power, groove angles, and weaving or rotate speed.

## 1. Introduction

Affected by the groove shape and position, contact tip to workpiece distance (CTWD) would change regularly in welding torch weaving or rotation processes, which can be reflected by the changes of arc signals. By sampling and analyzing the arc signal variation, the welding torch position in grooves can be monitored and controlled. This is the weld seam tracking technology based on arc sensing (arc tracking technology, for short). Taking welding arc signals as sensing signal directly, the sensor and the welding arc are coincident and the interference of wire bending and magnetic blow have been taken into account at the same time. Thus, the arc sensor has advantages of simple structure, resisting arc light interference, good real-time performance, and has attracted extensive attention from the beginning of the last century in 80 s. People have established the static and dynamic mathematical models and studied methods to improve accuracy and reliability of arc sensors. For example, Halmoy has analyzed the relationship between the power supply, the wire stickout and the arc characteristics and has built up a mathematical model for the high speed rotational arc sensing system in GMAW process. They also pointed out that the initial heat capacity must be considered when calculating the stickout voltage [1]. Kim and Na studied the effect of stickout, welding current and gas composition on the wire melting rate and found out that the energy melting the wire was composed of the arc energy and the resistance heat. They built a model for arc sensing taking the shape of welding pool into account [2,3]. Shi et al. has set up a kinematics model of the wire end for T joint and obtained an arc sensing model for GMAW process [4,5]. Ushio has developed a nonlinear model to describe the relationships between the output (welding current and voltage) and the input (torch height) of arc sensor in DC MAG welding in open arc mode, and a linearized model has also been derived from the nonlinear model under the hypothesis of any variable only showing a smaller variation around a given operating point [6,7].

All of the above models are assumed to have internal resistance and internal inductance and the power supply has a characteristic of constant current or constant voltage, but for a P-GMAW power supply, the internal parameters are switched quickly with the pulse frequency to ensure it works on two different external characteristic curves, and the above methods for building a mathematical model are not applicable any more. At present, the models used in P-GMAW sensing are all empirical models by fitting experimental data. Kim et al. have studied the P-GMAW arc morphology and current characteristics when groove angle was 45° and 60°. They analyzed the laws of arc characteristics when groove angle getting smaller, which have laid a technical foundation for the P-GMAW arc sensing technology [8,9]. Arc sound energy distribution of narrow gap P-GMAW has been studied by Hu et al., and they found that the arc sound spectrum was highly correlated with the arc position in groove [10]. Ye et al. studied the signal characteristics and processing methods of sensing torch height in P-GMAW process with an arc sensor [11]. Hyeong-Soon analyzed the sensitivity of current and voltage to torch height changing. By taking voltage signal as tracking signal and using moving average filtering method, a dual torch automatic welding system for narrow gap welding was designed [12,13]. In this paper, based on the characteristics analysis of P-GMAW power, the static mathematical model of P-GMAW arc tracking sensor was established, the key assumptions were studied, and the linear feature was verified through experiments.

## 2. P-GMAW Power Arc Length Control Method

At present, most commercially available P-GMAW welding power supplies adopt an I/I control mode, in which both the pulse current and the base current are held constant. The arc length variation does not directly affect the welding current value. In order to regulate arc length and ensure the arc stability, arc voltage-which is roughly proportional to arc length-is typically monitored and compared to the reference voltage. When an arc voltage change is detected, the pulse frequency is changed by adjusting the base current over time. The average current and the wire melting speed are changed to regulate the arc length because the pulse current *I_p_* and pulse time *T_p_* are invariant and the base current duration *T_b_* has changed. In this manner, a P-GMAW power supply can adjust arc length without changing the “one drop per pulse” transition. In modern arc welding power supplies, an arc length control model-where welding current compensation and pulse frequency are adjusted at the same time-is used to ensure that the arc length can be recovered in a short time and guarantee welding quality. The P-GMAW arc sensing mechanism is based on the P-GMAW arc length control mechanism.

## 3. Linear Mathematical Model for the P-GMAW Arc Tracking Sensor

**Figure 1:**
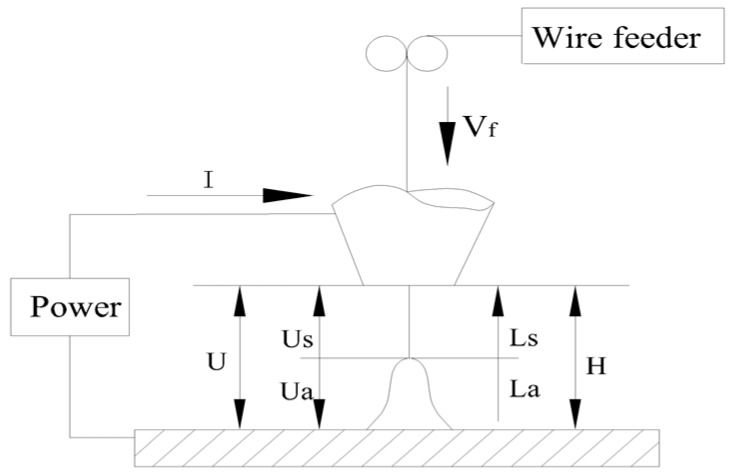
Monitored voltage composition.

As shown in Figure 1, CTWD is expressed in *H*, and the monitored voltage, *U*, is the sum of the arc voltage, *U_a_*, and the stickout voltage, *U_s_*, that is:

(1) $$U=U_{a}+U_{s}$$

According to the Ayrton equation [14]:

(2) $$U_{a}=U_{0}+IR_{a}+(E_{al}+E_{ai}I)L_{a}$$

(3) $$U_{s}=k_{s}L_{s}I$$

where *U*_0_ is the sum of the cathode voltage drop and the anode voltage drop, *R_a_* is the welding arc equivalent resistance, *k_s_* is the stickout resistance per unit length, *E_al_* and *E_ai_* are the arc length influence coefficients—which are equivalent to the field strength and resistance per unit length respectively and associated with the shielding gas and welding wire, *L_a_* is the arc length, *L_s_* is the welding stickout length, and I is the welding current.

**Figure 2:**
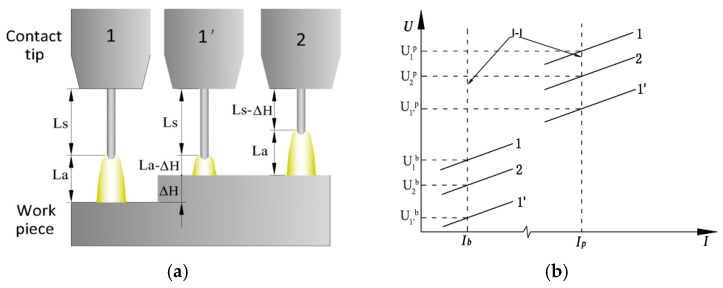
Voltage signal when the CTWD decreases: (**a**) arc length and stickout variation; (**b**) voltage changing process. The superscript *p* represents the pulse voltage and *b* represents the base voltage.

As shown in Figure 2, at the moment that the CTWD suddenly decreases, the arc length becomes shorter and the stickout remains unchanged, which results in the monitor voltage reducing to U_1_^’^ (base voltage is marked as U_1_^’b^ and the pulse voltage is marked as U_1_^’p^ in Figure 2b). Assuming that the welding power supply has excellent arc length control ability, which means that the time to restore the original arc length is much less than the torch weaving cycle time, the arc length should be recovered quickly by adjusting the pulse frequency. After adjusting, the change in the monitored voltage, *U*_2_, with respect to the *U*_1_ is related only to the stickout change, *L_s_*, that is:

(4) $$\Delta U=U_{1}-U_{2}=\Delta U_{s}=k_{s}I\Delta L_{s}$$

As shown in Figure 2a, the CTWD is composed of arc length *L_a_* and stickout *L_s_* and because *L_a_* is unchanged after adjusting, the CTWD variation equals the stickout variation, namely Δ*H* = Δ*L_s_*. So:

(5) $$\Delta H=\frac{\Delta U}{k_{s}I}$$

That is to say, the CTWD variation—which results from the stickout change—is reflected by the monitored voltage variation. In case of the stickout length plays no effect on, the CTWD variation will be proportional to the voltage change. Similar analysis can be done when the CTWD increases suddenly. On the other hand, in stable state 2 when the adjustment has finished, the wire melting rate, *V_m_*, should still equal the wire feed speed, *V_f_*, that is:

(6) $$V_{m}=k_{1}I_{av}+k_{2}L_{s}I_{av}^{2}=V_{f}$$

Namely:

(7) $$L_{s}=\frac{V_{f}-k_{1}I_{av}}{k_{2}I_{av}^{2}}=\frac{V_{s}}{k_{2}I_{av}^{2}}-\frac{k_{1}}{k_{2}}\frac{1}{I_{av}}$$

where *k_1_* and *k_2_* represent the melting rate coefficients. *I_av_* is the average welding current. The base current, *I_b_*, is very low, so the welding wire melting and droplet transfer mainly result from the pulse current, *I_p_*, and pulse duration, *T_p_*. Therefore, the average current *I_av_* in Equation (6) can be approximately expressed as:

(8) $$I_{av}=\frac{T_{p}I_{p}}{T}=T_{p}I_{p}f$$

Substituting Equation (8) into Equation (7), we obtain:

(9) $$L_{s}=\frac{V_{f}}{k_{2}T_{p}^{2}I_{p}^{2}}T^{2}-\frac{k_{1}T}{k_{2}T_{p}I_{p}}=\alpha T^{2}-\beta T$$

and:

(10) $$H=La+\alpha T^{2}-\beta T$$

Therefore, the CTWD variation can also be monitored through the pulse period, *T* (or frequency *f*), that is:

(11) $$dH=dL_{s}(T)=L_{s}^{\prime }(T){|}_{T}\cdot dT=2\alpha TdT-\beta dT$$

**Figure 3:**
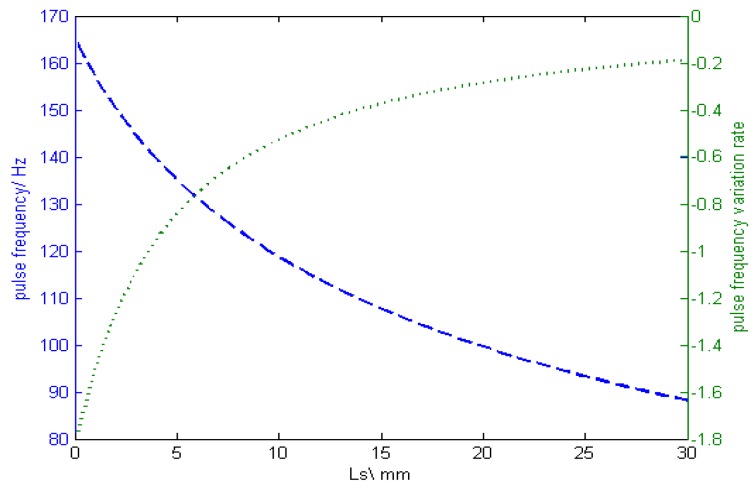
Stickout effect on pulse frequency.

In the formula:

$$\alpha =\frac{V_{f}}{k_{2}T_{p}^{2}I_{p}^{2}}$$

$$\beta =\frac{k_{1}}{k_{2}T_{p}I_{p}}$$

where *I_p_* = 500 A, *T_p_* = 2.5 ms, *V_f_* = 100 mm/s, and k_1_ and k_2_ come from the literature [7], the relationship between the stickout and pulse frequency is shown in Figure 3. 

When *L_s_* is larger, the pulse frequency variation rate tends to be constant, which indicates that the pulse frequency and *L_s_* follow a linear law. What’s more, the greater the initial CTWD, the more obvious the linear relationship. According to the above analysis, in order to ensure that the arc voltage and the pulse frequency are linearly related to the CTWD, we should reduce the arc length variation influence such that the CTWD change is a result of stickout change alone. For this purpose, the welding power requires excellent arc length control ability and a short welding arc combined with a longer stickout should be applied to the welding process.

## 4. Analysis on the Assumptions

Two important assumptions were used in previous model. First, we assumed that the welding power supply has excellent arc length control performance, and when the CTWD changes, the arc length can be quickly restored. Second, *k_s_* is regarded as constant so that the linear relationship between CTWD variation and monitoring voltage can be established. The first assumption directly affects the static model correctness, and the second assumption defines the relationship between CTWD variation and monitoring voltage.

### 4.1. Investigation of P-GMAW Power Arc Length Control Performance

**Figure 4:**
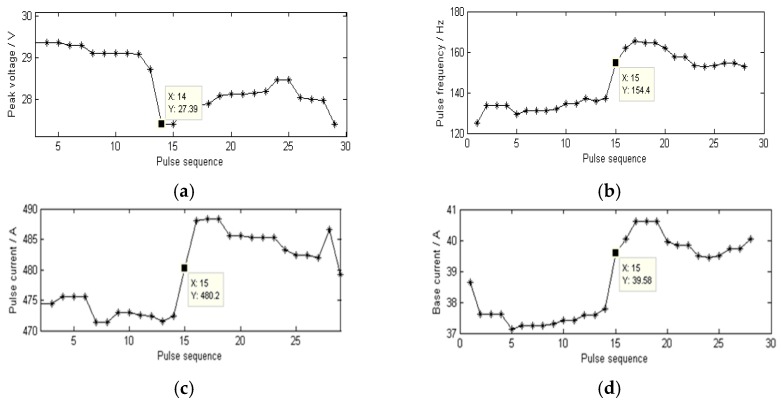
TPS3200 arc length control characteristics: (**a**) Pulse Voltage; (**b**) Pulse frequency; (**c**) Pulse current; (**d**) Base current.

Although welding power supplies from various manufacturers present different performances, the development of digital technology means that most products can meet the requirements for arc length control ability in the static model. As shown in Figure 4, the arc signals were sampled form a step test of P-GMAW using a TPS3200 welding power supply (Fronius, Pettenbach, Austria). The acquisition card collected the current and voltage data with a sampling frequency of 40 kHz. And, in each pulse cycle, the average value of the currents higher than 500 A was regarded as the pulse currents, and the voltage values corresponding to these current values were used to calculate the peak voltages. The average value of the currents lower than 80 A was regarded as the base current. The maximum current during each pulse cycle were obtained and the time intervals were calculated to get the pulse frequency. As the CTWD decreased suddenly by 4 mm, so the pulse voltage first decreased, and then after one pulse cycle, the peak current, the base current, and the pulse frequency are all follow the adjustment. The adjustment is completed within five pulse cycles—namely about 30 ms—which shows that the welding power supply can restore the arc length control within 30 ms when onto a 4 mm step suddenly. 

**Figure 5:**
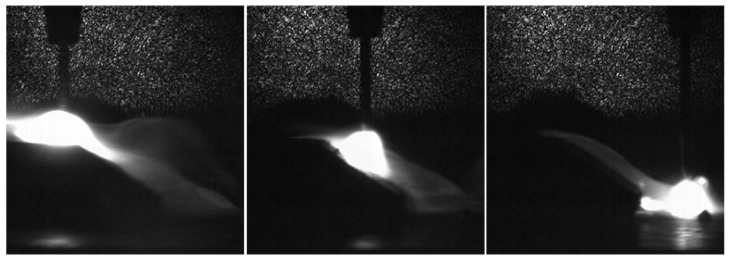
Arc images captured through high speed photography.

Figure 5 shows the arc shape when the torch moves towards a 45° slope at a 5 mm/s speed. Although the CTWD changes rapidly in the uphill process, the arc length is almost unchanged and CTWD variation is mainly affected by the stickout change. Through the above analysis we can determine that the arc length adjustment is fast and the arc is stable when the CTWD changes, which fully meets the requirements for deriving the mathematical model.

### 4.2. Stickout Effect on k_s_

The resistance of the unit stickout, *k_s_*, is influenced by the wire diameter, material, and temperature distribution. The wire diameter and material are determined under certain welding conditions, so *k_s_* varies only with the temperature distribution. Compared to the analytical method, the finite element method fully considers the thermo-physical material properties, so the results are more accurate. Therefore, the stickout temperature distribution was calculated through the finite element method.

#### 4.2.1. Stickout Temperature Field Modeling

(1) Control equations 

**Figure 6:**
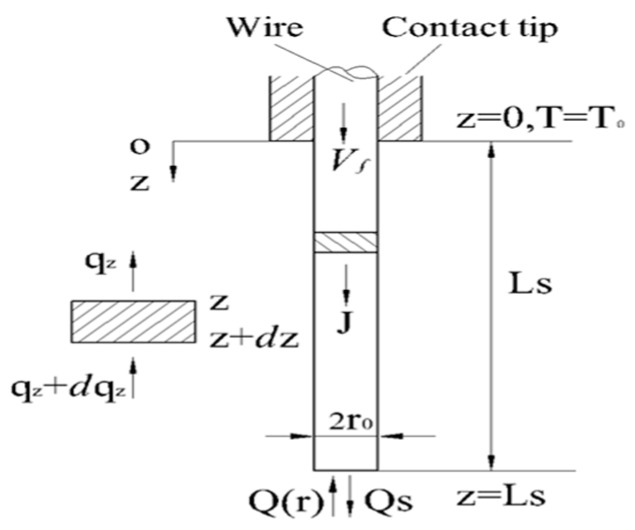
Modeling of stickout temperature field.

As shown in Figure 6 assuming that the wire feed rate, *V_f_*, equals the melting speed, *V_m_*—the welding wire is in a stable combustion state and the stickout, *L_s_*, remains constant. The welding wire temperature field equation can be expressed as:

(12) $$\rho C_{p}(\frac{\partial T}{\partial t}+V_{f}\frac{\partial T}{\partial t})=\frac{\partial }{\partial z}(\lambda \frac{\partial T}{\partial z})+J^{2}\rho_{c}$$

where *ρ_c_* is the electrical resistivity, λ is the thermal conductivity, and *J* is the current density.

(2) Boundary conditions

For the metal welding wire, the resistivity decreases as the temperature decreases. The temperature near the contact tip is much lower than other position along the wire, so the resistivity is assumed constant at *z* = 0 whenever stickout changes. This is described in the following boundary condition at the position *z* = 0:

(13) $$T{/}_{z=0}=T_{0}$$

where *T*_0_ is a constant temperature. 

At the end of the wire *z* = *L_s_*, the heat obtained through the welding arc within *dt* can be expressed as:

(14) $$dQ_{r}=q(r)Adt$$

where *q*(*r*) is the welding arc heat flux and *A* is the wire cross-section area. The heat lost through the melting wire and transition within time *dt* can be expressed as:

(15) $$dQ_{s}=\rho C_{p}V_{f}A(T_{m}-T_{0})dt+Q_{l}\rho V_{f}Adt$$

where *T_m_* is the melting temperature and *Q_l_* is the latent heat of fusion. The heat flux at the stickout end is:

(16) $${\lambda \frac{\partial T}{\partial z}/}_{z=Ls}=\rho C_{p}V_{f}(T_{m}-T_{0})+\rho V_{f}Q_{l}-q(r)$$

The wire surface heat flux of the heat distributing to the surrounding environment can be expressed as:

(17) $${\lambda \frac{\partial T}{\partial r}/}_{r=r_{0}}=h(T_{v}-T)$$

where *h* is the convective heat transfer coefficient, indicating the heat transfer capacity between the stickout surface and the surrounded air, and *T_v_* is the environment temperature. 

Therefore, the stickout temperature equation with a feed rate, *V_f_*, can be written as:

(18) $$\left\{ \begin{array}{l} \rho C_{p}(\frac{\partial T}{\partial t}+V_{f}\frac{\partial T}{\partial t})=\frac{\partial }{\partial z}(\lambda \frac{\partial T}{\partial z})+J^{2}\rho_{c}\\ T{/}_{z=0}=T_{0}\\ {\lambda \frac{\partial T}{\partial z}/}_{z=Ls}=\rho C_{p}V_{f}(T_{m}-T_{0})+\rho V_{f}Q_{l}-q(r)\\ {\lambda \frac{\partial T}{\partial r}/}_{r=r_{0}}=h(T_{v}-T) \end{array}\right.$$

#### 4.2.2. Solution and Analysis

**Figure 7:**
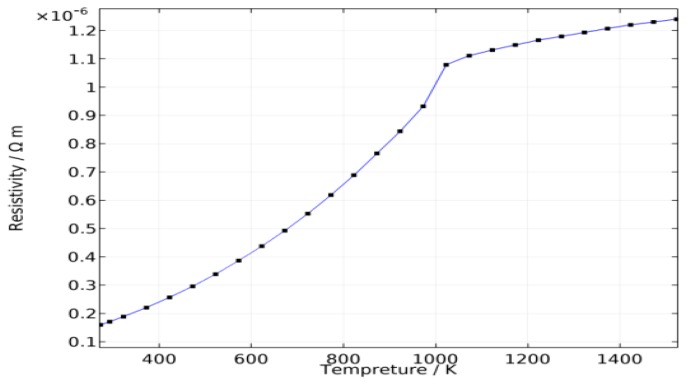
Carbon steel wire resistivity.

**Figure 8:**
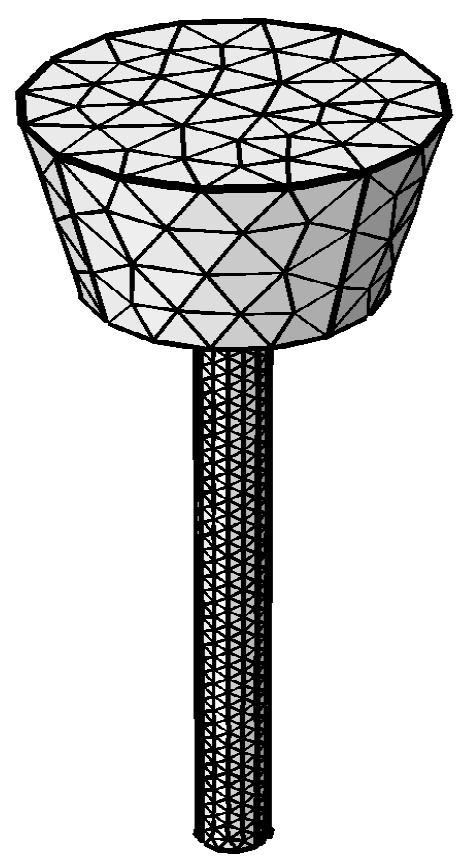
Three-dimensional model and mesh.

The partial differential equations were solved using the COMSOL software. The wire material thermo-physical properties were given according to carbon steel properties in the literature [15] and the wire resistivity is a function of temperature, as shown in Figure 7. For wire diameter *r*_0_ = 1 mm and feed rate *V_f_* = 100 mm/s, the average welding currents are 129 A, 118 A, 112 A, 105 A, and 100 A when the CTWDs are 15 mm, 17 mm, 19 mm, 21 mm, and 23 mm, respectively, which are determined through experiments. The arc length in the experiment is about 3 mm, and hence the stickout, *L_s_*, are 12 mm, 14 mm, 16 mm, 18 mm, and 20 mm. The three-dimensional model and mesh are established as shown in Figure 8.

**Figure 9:**
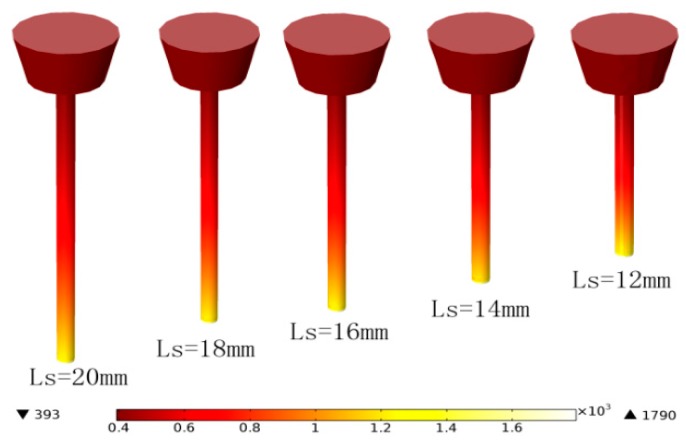
Wire temperature distribution.

**Figure 10:**
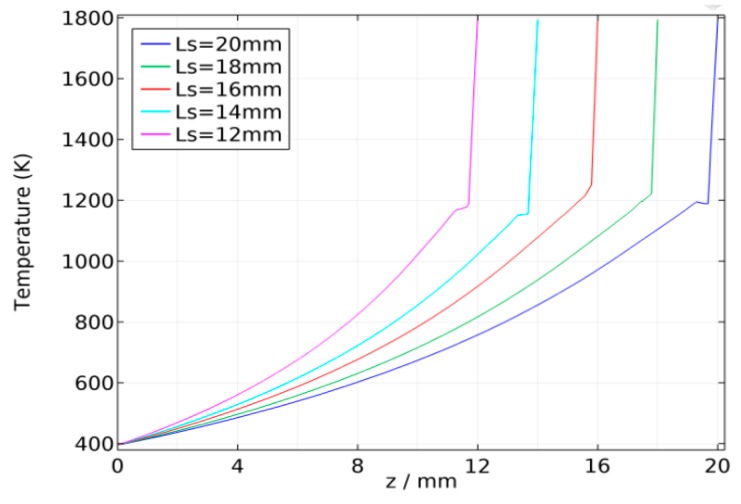
Temperature distribution along the wire.

**Figure 11:**
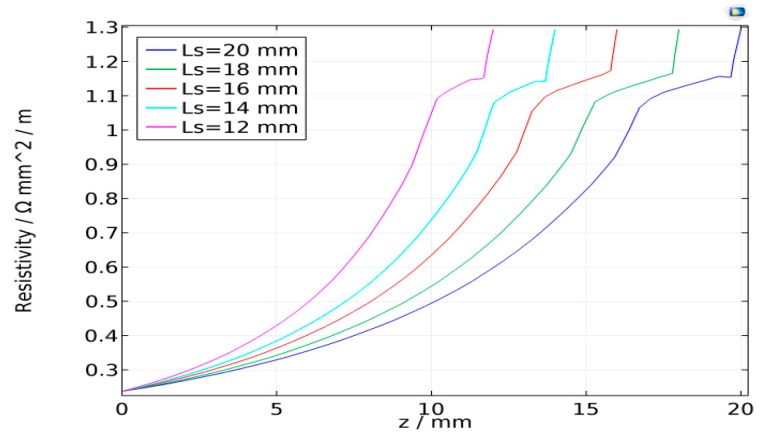
Resistivity distribution along the wire.

The temperature distribution along the wire is shown in Figure 9 and Figure 10. Note that no matter how the torch height changes, the temperature rises rapidly to the melting temperature at about 1 mm from *z* = *L_s_*, while the temperature distribution elsewhere in the wire is much more gradual. The resistivity along the stickout shows a similar distribution, as shown in Figure 11. The average welding wire resistivity with different stickout length were calculated and are listed in Table 1. From Table 1, when the CTWD increased from 15 mm to 23 mm, the average resistivity was approximately 0.6 (Ω mm²/m). The effect of CTWD variation on the unit stickout resistance, *k_s_*, is not obvious. Therefore, it is feasible to deal with the *k_s_* as constant in Formula (5).

## 5. Experimental Verification of Linear Features in the Mathematical Model 

### 5.1. Experimental Conditions

According to the previous analysis, the voltage and pulse period *T* (or frequency *f*) can reflect the CTWD change in P-GMAW process. The following work is to experimentally verify the linear characteristics. 

**Figure 12:**
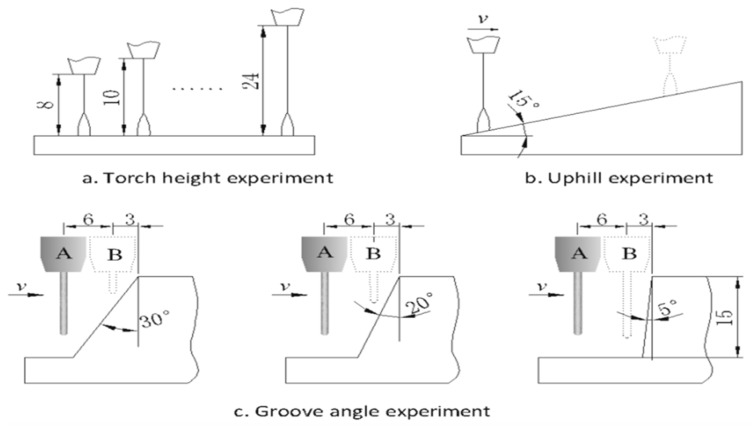
Experimental schemes.

The experimental parameters are shown in Table 2. The welding power supply is a Fronius TPS3200 operated in P-GMAW mode; the wire feed rate is fixed and other parameters are automatically matched. The experimental scheme is shown in Figure 12. First, we analyzed the influence of different CTWD on the arc signals through a plate welding experiment, in which the CTWD is changed by shifting the torch height. Then we analyzed the influence of continuously changing the CTWD on arc signals with an uphill experiment. Finally, the arc characteristics for different groove angles are verified.

### 5.2. Arc Signal Characteristics When Changing the Torch Height

**Figure 13:**
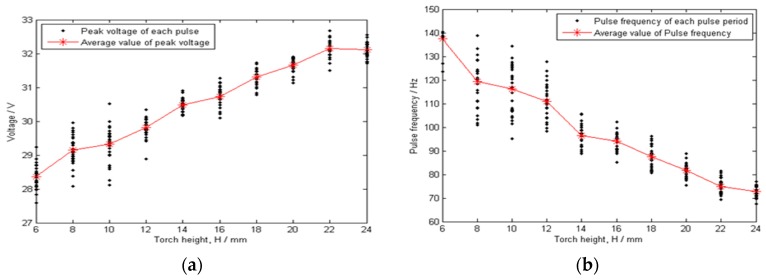

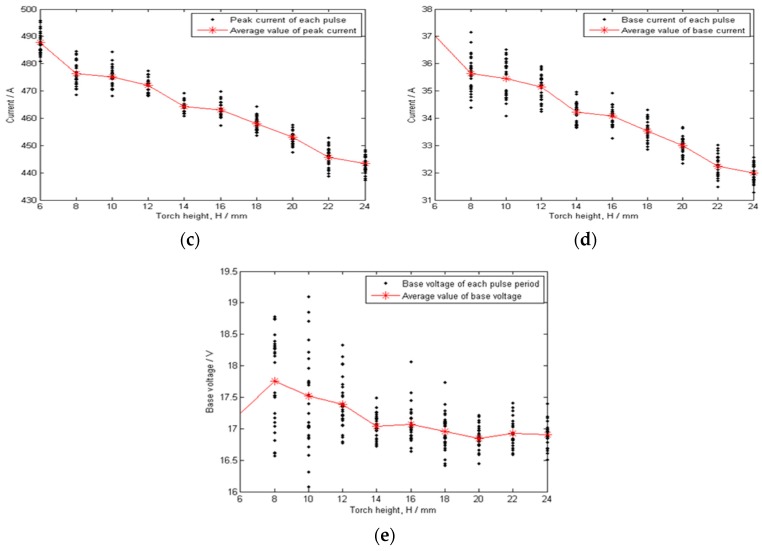
Arc signal characteristics for different torch heights: (**a**) Pulse voltage; (**b**) Pulse frequency; (**c**) Pulse current; (**d**) Base current; (**e**) Base voltage.

We examined the relationship between the torch height and arc signals—such as pulse voltage, base voltage, pulse current, base current, and pulse frequency—in the plate welding process, which is shown in Figure 13.

In Figure 13, the pulse current and pulse voltage are the average values during each pulse period, T_p_, and the base current and base voltage are the average values during the base period, T_b_; the pulse frequency is obtained by calculating the pulse intervals. The pulse intervals are marked with black dots. The arc parameter average values (e.g., pulse current, pulse voltage, base current, base voltage, and frequency) for all pulse cycles in a given torch height are marked with asterisks. Note that the arc parameters—except base voltage−are roughly linearly related to the torch height. Also, the shorter the torch height, the greater the data fluctuation.

**Figure 14:**
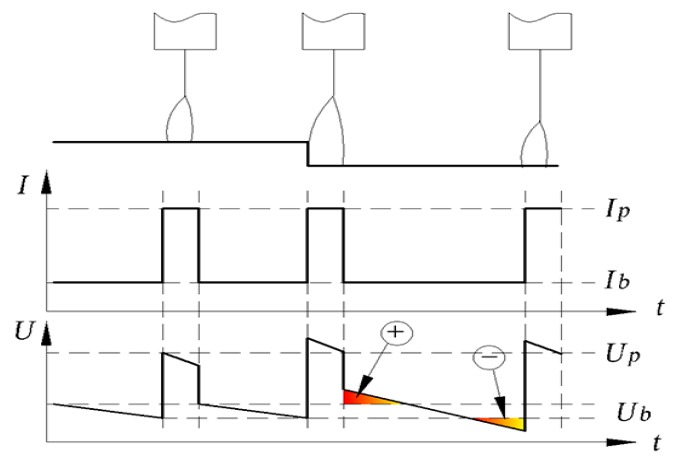
Base voltage when torch height becomes larger.

As shown in Figure 13e, the linear characteristic between the base voltage and the torch height is not obvious. Even though the base voltage increases when torch height increases, the arc length gets shorter and the voltage decreases during the base period late stage due to the increased base period and continuous wire feed rate; these offset the voltage increase resulting from the torch height increase, as shown in Figure 14. The situation is similar to the scenario where the torch height decreases. In addition, because the base current is small, the arc is easily disturbed and the arc voltage is not stable. Therefore, the base voltage is not suitable for use as the seam tracking characteristic parameter.

### 5.3. The Influence of Continuously Changed CTWD on Arc Signals

**Figure 15:**
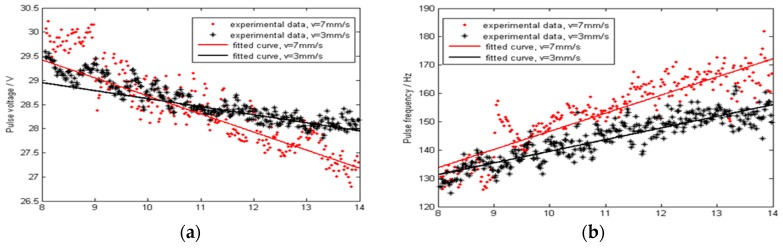

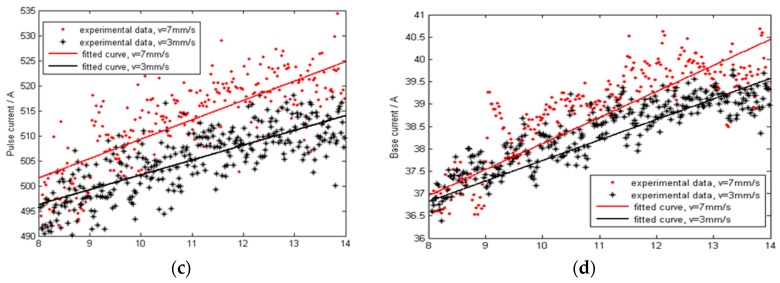
Effects of welding speed on the arc signal when uphill welding: (**a**) Pulse voltage; (**b**) Pulse frequency; (**c**) Pulse current; (**d**) Base current.

As shown in Figure 15, the arc signal changes linearly with the CTWD in the uphill process, and the pulse voltage and frequency exhibit a better linear relationship in comparison to the base and pulse current. In addition, when the welding speed is increased, the characteristic signal sensitivity (slope) also increases.

### 5.4. Influence of Groove Angle on Arc Signal

**Figure 16:**
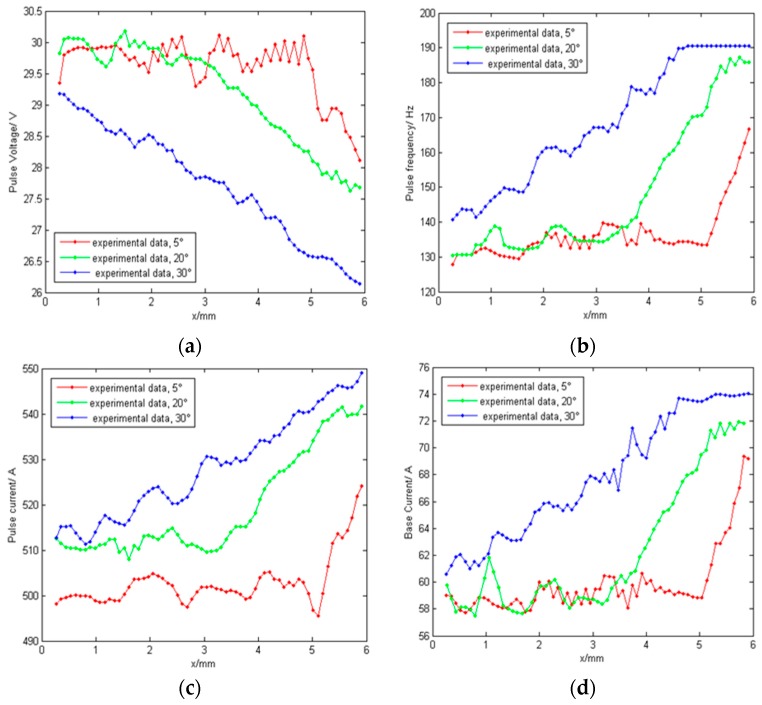
Arc signals at different groove angles: (**a**) Pulse voltage; (**b**) Pulse frequency; (**c**) Pulse current; (**d**) Base current.

As shown in Figure 12c, the torch moves from position A to the side wall B; the groove angles are 30°, 20°, and 5°, respectively. The arc parameter variations in response to torch movement are shown in Figure 16. As the torch moves towards the side wall, the arc voltage linearly decreases while the current and the pulse frequency linearly increases (in 30°, 20°, and 5° order). Relative to the large groove angle (α = 30° and α = 20°), when the groove is small (α = 5°), the characteristic parameter variations are small, but the variation rates (slope) are greater.

## 6. Conclusions

Based on P-GMAW welding power supply characteristic analysis with an I/I model, the behavior governing arc voltage and pulse frequency in response to torch height change are derived under the assumption that the welding power supply has excellent arc length control performance. Neglecting the influence of stickout on unit resistance, *k_s_*, CTWD variation is proportional to voltage variation, and the pulse frequency is also roughly linearly related to the CTWD when the torch height is large.

By verifying the arc length control characteristics for a TPS3200 welding power supply, the results show that the arc length control ability assumption is feasible. The temperature distribution and resistivity regularity along the stickout are calculated through a Three-dimensional finite element model. The calculation results show that CTWD variation does not affect the average resistivity, and it is feasible to ignore the stickout resistance variation.

The welding experiments show that the P-GMAW arc signals all satisfied the linear relationships. In addition, the faster the welding speed, the higher the arc signal sensitivities; the smaller the groove angle, the greater the arc sensitivity. The arc signal variation rates need to be modified according to the specific welding requirements.

## Figures and Tables

**Table 1:** The average resistivity for different stickout.

Stickout/mm	12	14	16	18	20
Average resistivity/Ω mm²/m	0.6	0.585	0.60	0.592	0.596

**Table 2:** Experiment parameters.

Parameters	Value
Welding speed/mm·s^−1^	5
Wire diameter/mm	1
Feeding speed/m·min^−1^	6
Shielding gas	80% Ar + 20% CO_2_
Base metal	Q235
